# Eag1 K^+^ Channel: Endogenous Regulation and Functions in Nervous System

**DOI:** 10.1155/2017/7371010

**Published:** 2017-03-06

**Authors:** Bo Han, Tursonjan Tokay, Guangming Zhang, Peng Sun, Shangwei Hou

**Affiliations:** ^1^Department of General Surgery, Tongren Hospital, Shanghai Jiao Tong University School of Medicine, Shanghai 200336, China; ^2^Hongqiao International Institute of Medicine, Tongren Hospital, Shanghai Jiao Tong University School of Medicine, Shanghai 200336, China; ^3^Center for Life Sciences, National Laboratory Astana and School of Sciences and Technology, Nazarbayev University, Astana 010000, Kazakhstan; ^4^Department of Anesthesiology, Tongren Hospital, Shanghai Jiao Tong University School of Medicine, Shanghai 200336, China

## Abstract

*Ether-à-go-go*1 (Eag1, Kv10.1, KCNH1) K^+^ channel is a member of the voltage-gated K^+^ channel family mainly distributed in the central nervous system and cancer cells. Like other types of voltage-gated K^+^ channels, the EAG1 channels are regulated by a variety of endogenous signals including reactive oxygen species, rendering the EAG1 to be in the redox-regulated ion channel family. The role of EAG1 channels in tumor development and its therapeutic significance have been well established. Meanwhile, the importance of hEAG1 channels in the nervous system is now increasingly appreciated. The present review will focus on the recent progress on the channel regulation by endogenous signals and the potential functions of EAG1 channels in normal neuronal signaling as well as neurological diseases.

## 1. Introduction

*Ether-à-go-go*1 (Eag1), a voltage-gated K^+^ (Kv) channel firstly identified in a leg shaking mutant phenotype of* Drosophila* caused by spontaneously repetitive action potential firing in motor neurons and increased transmitter release [1, 2], is conserved in diverse mammalian species including human. Three subfamilies of the mammalian homologs, EAG, ERG (eag-related), and ELK (eag-like), have been identified [3] and show distinct expression patterns and electrophysiological properties [4]. Compared to the well-known function of human ERG1 (HERG1) channels in long QT syndrome in cardiac arrhythmia, the physiological roles of EAG channels remain largely unknown [5]. So far, two EAG isoforms Eag1 (KCNH1, Kv10.1) and Eag2 (KCNH5, Kv10.2) have been identified and they show similar electrophysiological features such as slow voltage-dependent activation, noninactivating, and inhibition by intracellular Ca^2+^  [Ca^2+^]_i_ [4, 6, 7]. Their responses to extracellular quinidine, a broad-spectrum K^+^ channel inhibitor, however, were noticeably different, indicating this compound can be used to distinguish the EAG channel subtypes in native cells [7]. In physiological conditions, both EAG1 and EAG2 channels are expressed in the brain and their distributions overlap in the cortex and olfactory bulb, but show some differential expression pattern in other specific locations such as thalamus [8]. The nonneural distributions of EAG1 channels are highly constricted to a large variety of cancer cells and their roles in cancer growth, metastasis, and the potential diagnostic and therapeutic significance have been well established [5, 9, 10]. Likewise, EAG2 channels, although less extensively studied, have also been revealed recently to play important roles in medulloblastoma development and to be a potential therapeutic target and a tumor marker [11, 12].

In humans, EAG1 is encoded by the KCNH1 gene located on chromosome 1q32.1–32.3 [4]. Four alternative transcripts have been identified in the human brain and they can translate into four different forms of proteins including the canonical (most abundant) form, a longer variant containing a 28-residue stretch between the transmembrane segments S3 and S4, and two shorter forms recently identified, respectively [13]. The stretch containing form shows no evident functional differences compared to the canonical full-length standard channel. By contrast, both shorter forms fail to form functional ion channels because of lacking all transmembrane segments but both can significantly reduce the current of the full-length form when coexpressed in* Xenopus* oocytes [13]. Like other Kv channels, the core region of EAG1 channel has six helical segments (S1 to S6), including the voltage sensor (S1–S4) and the K^+^-selective pore (S5, pore helix, and S6). Even though the overall architecture of the EAG1 channels is similar to that of previously crystallized Kv channel structures, there are many different aspects in S2-S3 linker, S4, and S4-S5 linker based on the structural models of rat EAG1 (rEAG1) channels derived by single-particle cryoelectron microscopy (cryo-EM) [14]. These local structural characters may determine that EAG1 channel has a fundamentally different voltage gating process compared to other types Kv channels. In addition, its intracellular domains are structurally distinct from other classical Kv channels in that a long N-terminal region contains an eag domain comprised of a Per-Arnt-Sim (PAS) domain and a PAS-cap domain, while the C-terminal region contains a cyclic nucleotide binding homology domain (CNBHD), which is connected to the pore through a C-linker region [15–17]. The CNBHDs of EAG1 channels share a high degree of sequence similarity with the cyclic nucleotide binding domain conserved in the cyclic nucleotide-gated (CNG) channels and hyperpolarization-activated cyclic nucleotide-modulated (HCN) channels [16]. However, CNBHD does not bind cyclic nucleotides [17–19]. The crystal structure of eag-CNBHD complex of mEAG channel has suggested that the coupling between eag and CNBHD is involved in EAG channel gating regulated by eag domain [17]. The recent cryo-EM structure of rat EAG1 channel has further clearly shown that the PAS domain is located at the periphery of the intracellular region and interacts primarily with the CNBHD from a neighboring subunit [14]. The S6 helix extends into the intracellular region and connects to the C-linker, which forms an intracellular ring directly above the CNBHD by which the C-linker couples the movements of the S6 and CNBHD [14].

The function of the EAG1 channels in nervous system remained elusive until recently. A recent series of studies using gene knock-out animals and electrophysiological recordings have provided strong evidence that EAG1 channels are important for the neuronal excitability regulation [20]. The clinical observations and genetic tests further revealed that the gain-of-function mutations of EAG1 channels are closely associated with two rare neuronal developmental diseases Zimmermann-Laband and Temple-Baraitser syndromes (ZLS and TBS) [21–24]. This article will briefly summarize the recent progress on the mechanisms of EAG1 channel gating regulation by endogenous molecules and discuss its physiological and pathological functions in nervous systems in mammals.

## 2. Endogenous Regulation of EAG1 Channels

**Figure 1:**
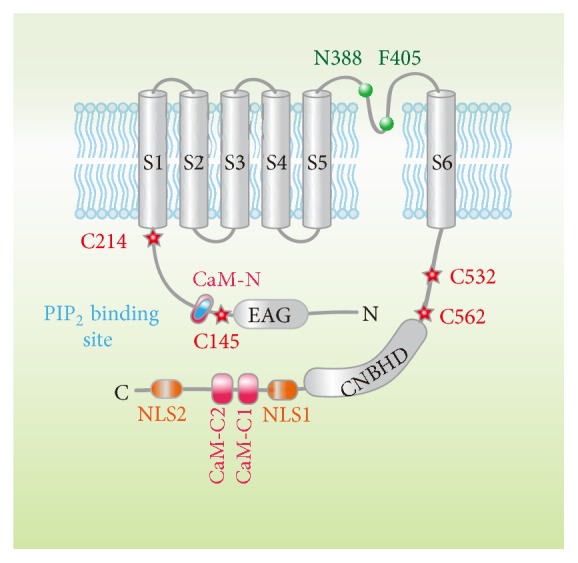
Schematic representation of a KCNH1 subunit (NM_002238.3) indicating a PIP_2_ binding site (blue), three CaM binding domains (magenta), two glycosylated positions (green balls), four oxidative modification sites (red stars), and two nuclear localization signals (NLS) (orange).

The function of EAG1 channel can be regulated by a large variety of proteins such as K^+^ channel regulator 1 (KCR1) [25], CaM-dependent kinase [26], 14-3-3 protein [11], epsin [27], rabaptin 5 [28], and cortactin [28]. These proteins function as the channel partners and affect the trafficking and expression of EAG channels. Additionally, the channels are acutely regulated by a large array of endogenous small molecules including Mg^2+^ [29], H^+^ [30], H_2_O_2_ [31], arachidonic acid (AA) [32], Ca^2+^ [33], phosphatidylinositol 4,5-bisphosphate (PIP_2_) [34], and hormones [35–37]. The EAG channels also undergo N-linked glycosylation before its insertion into plasma membrane [38]. Recent report has shown that nuclear localization signal (NLS) can regulate the localization and function of EAG protein [39]. The functions and potential mechanisms of regulation by most of the aforementioned protein factors have been reviewed recently [5]. In this review, we will discuss the EAG channel regulation by H_2_O_2_, Ca^2+^, PIP_2_, glycosylation, nuclear localization signal (Figure 1), arachidonic acid, and hormones mainly focusing on the recent mechanistic progress.

### 2.1. Redox Modification

Both the expression and the function of EAG1 channels are linked to cellular oxygen homeostasis. Downie et al. [40] reported that EAG1-expressing tumors showed increased angiogenesis compared to the EAG1-negative control. EAG1 channel was proposed to interfere with hypoxia homeostasis by increasing the basal HIF-1*α* activity and lowering the threshold of HIF-1*α* activation by hypoxia. Mild hypoxia (5% O_2_) induced HIF-1 expression in EAG1-expressing cells while no increase in HIF-1 was observed under normoxia. Sahoo et al. [31] observed that ionic currents through the heterologously expressed EAG1 channels are progressively inhibited by intracellular application of H_2_O_2_, an oxidative stress mediator, possibly via attacking multiple cysteine residues as revealed by a systematic study on channel modification by specific cysteine modifying agents such as DTNB, MTSES^+^, and MTSET^−^ combined with cysteine mutations and electrophysiological assays. They found that the kinetics of cysteine modification of EAG channels contains two components; a rapid component resulted in an alteration of gating parameters of half-activation voltage, activation and deactivation kinetics, and the channel open probability while the slow component caused a complete loss of channel function. The fast component is mediated by two cysteine residues (C145:C214) in the linker connecting to S1, while the slower component strongly depends on two cysteines in the C-linker region (C532:C562) connecting to the channel gate/pore (Figure 1).

### 2.2. Ca^2+^/CaM

Intracellular Ca^2+^  [Ca^2+^]_i_ inhibits EAG1 channels with a half-maximal inhibition at a concentration of ~100 nM [33] and the inhibition requires a direct binding of CaM [41, 42]. A peptide screening combined with electrophysiological recording has revealed three discrete sites (BD-C1: 674–683, BD-C2: 711–721, and BD-N: 151–165) potentially binding to Ca^2+^/CaM (Figure 1). Mutation of any of these domains can impair the inhibition of hEAG1 currents by Ca^2+^ [41]. Fluorescence correlation spectroscopy (FCS) affinity assay further indicated that CaM binding to BD-C1 is of lower affinity as compared to the other two sites [41]. Consistently, by visualizing the interaction between YFP-labeled CaM and Cerulean-labeled hEAG1 in mammalian cells by Förster resonance energy transfer (FRET), Gonçalves et al. [43] found that the high affinity BD-N binding domain and the second C-terminal binding domain BD-C2 are predominantly involved in EAG1 channel inhibition by Ca^2+^. Consistently, a recent isothermal titration calorimetry (ITC) combined with crystal structure of the CaM-BDC2 complex study revealed a high affinity between CaM and BD-C2 [44]. Mutations at these two sites completely abolished CaM binding to hEAG1, indicating that the BD-N and BD-C2 binding domains are required for CaM binding. The recent CaM-bound cryo-EM structure model of rat EAG1 channels provided more detailed information [14]. It is clearly shown that the channel pore becomes smaller than the diameter of hydrated potassium (6 to 8 Å) when CaM is bound to the channel. The further inspection of CaM-bound EAG1 structure showed that all three previously identified CaM binding sites are occupied by one CaM and four CaM molecules are bound to the functional tetramer channel. The two contact regions in the C terminus of CNBHD form helices that act as a claw to grab the CaM C-lobe. The first helix (residues 683 to 695) of the lower affinity BD-C1 domain interacts with the top of the CaM C-lobe near the Ca^2+^ binding sites and the second helix (residues 708 to 722) of the higher affinity BD-C2 domain is bound to the hydrophobic pocket of the CaM C-lobe. The hydrophobic core of the CaM-N-lobe binds to a helix (residues 147 to 157) of high affinity BD-N on the PAS domain. Based on the structure model, the authors hypothesized that once [Ca^2+^]_i_ increased, CaM may act as a molecular clamp to pull the PAS domain and CNBHD together and then alter the orientation of neighboring CNBHD. The orientation alteration will lead to a rotation of both the C-linker and S6, thereby constricting the intracellular gate and to close the channel pore. In agreement with the structural findings, the functional study by Lörinczi et al. [45] showed that deletion of the eag domain, CNBHD, or the C-linker abolished the inhibitory of [Ca^2+^]_i_ on EAG1 channels. All these structural and functional findings confirmed that eag domain and CNBHD interaction and the downstream conformational changes in C-linker region are critical in Ca^2+^/CaM-induced EAG channel gating inhibition.

### 2.3. PIP_2_

PIP_2_, a phospholipid composed of one negatively charged head group and two fatty acid tails, serves as a structural cofactor for many membrane proteins and it is also the precursor of two important second messengers, diacylglycerol (DAG) and inositol 1,4,5-trisphosphate (IP_3_) [46]. It is well known that PIP_2_ modulates a large variety of ion channels, in which PIP_2_ either works as a direct channel gate opener or as a current enhancer by facilitating the gating process [47]. Interestingly, our previous studies have shown that PIP_2_ functions as a potent inhibitory gating modifier of the hEAG1 channel by shifting the overall voltage dependence of activation to the positive direction [34]. Although not accurately measured, the voltage dependence shift caused by PIP_2_ may be >100 mV based on the observation that a functionally saturating concentration of PIP_2_ (3 *μ*M) essentially eliminates the current at 40 mV while the normalized conductance without PIP_2_ is near unity. In addition to PIP_2_, other types of phosphatidylinositol (PIs), such as PI, PI(4)P, PI(3,4,5)P_3_, and PI(3,5)P_2_, also differentially alter the hEAG1 current, from no effect by PI to the strongest inhibition by PI(3,5)P_2_, suggesting that the number of negative charges of the head group is also critical for the channel inhibition. By using biolayer interferometry (BLI) technique, a novel methodology for detecting protein-protein or protein-small molecules interactions [48], we measured the kinetics change of binding between PIP_2_ and purified hEAG1 channel protein and found that the kinetics of the BLI signal resembled those observed in the patch clamp measurements in the excised patches. In addition, the dissociation constant (*K*_*d*_) value derived from BLI measurement is about 0.35 *μ*M, similar to the IC_50_ value obtained from the electrophysiological study. The PIP_2_ inhibition of hEAG1 channels is physiologically relevant as evidenced by the observation that neuronal transmitter 5-HT noticeably increased the whole-cell current from the hEAG1 channels coexpressed with the serotonin receptor HTR_2A_ in HEK293 cells, suggesting that endogenous PIP_2_ may exert a detectable tonic inhibitory influence on hEAG1 channels in intact neurons. PIP_2_ also modifies other members of EAG K^+^ channel family. For instance, PIP_2_ increases currents through hERG1 possibly via a short region rich in positively charged residues (^883^RQRKRKLSFRRR^894^) in the intracellular C terminus [49]. By contrast, it inhibits hELK1 channels expressed in* Xenopus* oocytes by interacting with some of the positively charged residues in the S4-S5 linker, S6, and eag domain [50]. Amino acid sequences of hEAG1, hELK1, and hERG1 show considerable similarity (~40%) and these residues involved in PIP_2_ action in ERG and ELK are not well conserved in hEAG1 channels, indicating PIP_2_ may use different mechanisms for EAG channel inhibition. As expected, our BLI and mutational study on the interaction between PIP_2_ and the mutant channels clearly showed that PIP_2_ binding required a short segment located in the downstream of the EAG domain close to S1 (Figure 1), a short region called CaM-N identified previously because of the potential Ca^2+^/CaM binding [41]. Although lacking more detailed information, our results suggested that, similar to the approach by which Ca^2+^/CaM inhibits EAG1 channels [45], PIP_2_ may alter the voltage dependence of activation by changing the physical coupling between eag domain and CNBHD [14, 17].

### 2.4. Glycosylation

*N*-linked glycosylation is a common posttranslational modification of membrane proteins including many types of ion channels. The changes in the glycosylation pattern may affect the surface charge, which could then cause abnormal trafficking and dysfunction of ion channel, leading to neurological disorders [51]. Although* N*-linked glycosylation is very common, some types of ion channels such as Kv1.6 and Kv2.1 are glycosylation deficient [52]. Napp et al. [38] found that expression of Eag1 in glycosylation deficient Lec1 cells or tunicamycin-treated CHO cells induced the localization of Eag1 to unidentified perinuclear structures, suggesting the correct glycosylation is necessary for both Eag1 channel trafficking to the plasma membrane and the proper function. The glycosylation sites of EAG channels include Asn-388 and Phe-405, two residues located in the extracellular loop between the S5 and S6 transmembrane domains (Figure 1). But they show noticeable differences in the glycosylation pattern; Asn-388 seems to undergo only core glycosylation, but more complex sugars are bound to Phe-405, shifting the apparent molecular mass of single subunit of EAG1 channel from 100 to 110 and 130 kDa, respectively, as assessed by Western blot analysis. Besides the correct channel folding, trafficking, expression, and normal function of EAG channels, the other distinct physiological significance of glycosylation may relate to the channel's lower sensitivity to some toxins, which display potent inhibition on other types of Kv channels. The recent cryo-EM structure model of rEAG1 channel has clearly shown that EAG1 channel has an extended extracellular turret, a distinct structural character compared to all previously identified Kv channel structures [14, 53]. The sugar chains on the glycosylation sites in the turret can prevent the binding of the toxins to the channel pore.

### 2.5. Nuclear Localization Signal

The classical nuclear localization signal (NLS) facilitates the trafficking of a cellular protein into the cell nucleus for precise regulation of the cellular process such as gene expression [54], cell cycle, and signal transduction [55]. Since the first NLS identified in SV40 large T-antigen [56], many proteins including ion channel proteins have been found to carry one or multiple NLSs [39, 57–59]. The identification of the functional NLSs in ion channel proteins would provide novel roles besides the traditional ion permeation function. For instance, the NLS in the* C. elegans *TRPV channel OCR-2 facilitates the accumulation of a synthetic cargo protein and the C-terminal cytosolic fragment of OCR-2 [57]. The NLSs of the intracellular anion channel protein 4 (CICL4) can mediate the channel itself to enter into the nucleus in response to cellular stress [58] and the NLS of *γ*-subunit of the epithelial Na^+^ channel (ENaC) could regulate the channel activity in a feedback manner [59]. Interestingly, Sun et al. also found two NLSs in the carboxy-terminal of* Drosophila* Eag80, an 80 kDa EAG splice variant containing both N- and C-terminal sequences but lacking all channel-forming transmembrane domains [39]. The Eag80 C-terminal contains two putative nuclear localization signals NLS1 and NLS2 (Figure 1), one putative nuclear export signal (NES), and binding domains for CaM and CaMKII. Although it does not produce an active ion channel, Eag80 activates a signaling cascade leading to altered cell architecture and NLS1 has been demonstrated to be necessary for such cell morphology alteration. Although the exact mechanism by which the splice variant carrying NLSs of EAG channel protein change cell structure still needs to be uncovered, the established relationship between morphologic changes of neuron cells and aging as well as sporadic Alzheimer's disease is likely linked to the regulatory role of these cell shape remodeling related splices on the neural circuit associated with neurodegenerative diseases [60]. Apparently, the Eag80 is distinct from the inner nuclear membrane located EAG channel because the latter's nuclear localization does not require the NLS and it shows electrical properties compatible with full-length EAG1 channels [61]. Recently, two mammalian orthologs of the Eag80 splice variants in* Drosophila* have been identified in multiple tumor cells and brain tissue [13]. They are E65 and E70 with the predicted protein size of 65 and 70 kDa, respectively. Similar to EAG80, both variants completely lack the transmembrane domains and produced cytoplasmic proteins without any channel function. Coimmunoprecipitation assay shows that both E65 and E70 interact with the full-length EAG1 channel and can downregulate EAG1 current when they are coexpressed with the full-length form [13]. Surprisingly, their mechanisms for the full-length EAG channel current regulation are different. E65 causes an overall reduction in K_V_10.1 protein level and also reduces single channel current, whereas E70 mainly affected the glycosylation pattern but without detectably affecting the electrophysiological parameters [13]. The interaction between the full-length EAG1 channel and the splice variants does not rely on the C-terminal tetramerization domain. However, the interaction between the short isoforms and the full-length channel would occur through other unknown interaction sites. Unfortunately, none of these splice variants could be detected as protein in native systems. Thus, more studies are required before understanding their physiological functions of these NLS contained splice variants.

### 2.6. Arachidonic Acid

Arachidonic acid (AA), a long-chain polyunsaturated fatty acid, serves as a major constitute of cell membrane of neuronal tissues [62, 63] and can be released as a second messenger during neurotransmission [64, 65]. Maintaining a sufficient level of AA is a key issue for the neurological health. AA deficiency is associated with many neuronal diseases such as Alzheimer disease [66], bipolar disorder [67], epileptic seizures, and depression [68]. Accumulating evidence suggests that the effects of AA are mediated by regulation of some types of ion channels [69]. In the nervous system, AA and its metabolites can modulate activities of multiple ion channels directly or indirectly, including tetrodotoxin-sensitive (TTX-S) and tetrodotoxin-resistant (TTX-R) sodium channels [70], voltage-gated Ca^2+^ and K^+^ channels [71–73], Ca^2+^-dependent K^+^ channels [74], and acid-sensing ion channels [75]. HEAG, as one of the voltage dependence K^+^ channels responsible for controlling neuronal excitability in the nervous system, has been identified as another AA-sensitive ion channel by Gavrilova-Ruch et al. [32]. They reported that AA can enhance hEAG channels activity by shifting the voltage dependence of activation to the negative voltage, resulting in enhanced human melanoma cell proliferation. The previous studies have suggested that AA may directly bind to the channel pore [76, 77], N-terminal [78], or C-terminal sites [79]. The evidence from the kinetic changes of EAG1 channels by AA has suggested that AA directly interacts with the EAG1 channel. Besides EAG1, AA also activates EAG2 and ERG1 channels, implicating a conserved mechanism involved in AA-mediated activation of the EAG channel family [32, 80]. The neurological significance of EAG1 regulation by AA is not yet known; however, based on the wide distributions of AA and EAG1 in the nervous system, it is reasonable to expect that modulation of EAG1 by AA could play important roles in neuronal signaling and excitability.

### 2.7. Estrogen and Progesterone

Both estrogen and progesterone display protective effects against some neurodegenerative disorders including stroke, traumatic brain injury [81–83], Parkinson's disease [84, 85], and other neurodegenerative diseases [86, 87]. Like other steroids, the effects of estrogen and progesterone are thought to be dependent on their genomic effect via activation of their nuclear receptors [88, 89]. However, the nongenomic effect of both hormones has been appreciated and, at least in part, is dependent on their direct regulation of select ion channels. For example, progesterone at micromolar concentrations directly blocks the voltage-dependent calcium channel that triggers neurotransmitter release to alleviate the neurotoxicity in the brain [90] and the therapeutic concentrations can block the voltage-gated potassium channel in striatal neurons in rats [89]. Similarly, estrogen can exert rapid regulation of potassium and calcium channels in neurons by activation of estrogen receptors to regulate the neuronal activities [88] and inhibit the epileptiform bursting activities in cultured hippocampal neurons by activation of the voltage-gated potassium channel Kv4.2 [91]. Estrogen and progesterone have been reported to upregulate the expression of EAG channel proteins in cervical cancer cells and normal cervical cells [35–37]. The identifications of EAG as estrogen- and progesterone-responsive ion channel give a strong hint that EAG1 channels would be involved in the hormone induced signal pathways. Undoubtedly, future studies are needed to determine if native neuronal EAG channels can be modulated by these two hormones and what roles, if any, they may have in mediating physiological functions of EAG channels in CNS.

## 3. Functions of EAG1 Channels in Normal Neuronal Signaling

The functional contribution of EAG channel to excitability regulation in* Drosophila* has been extensively studied and well accepted in the field [1, 92–94]. In zebrafish, Stengel et al. found that EAG1 is crucial for the early embryonic development and patterning [95]. In mouse, the present evidence from EAG1 knock-out experiment has shown that EAG1 deficiency does not significantly alter the embryogenesis, development, social behavior, learning and memory, and the major electrical properties of cerebellar Purkinje cells. The deficiency of EAG1 only caused a mild hyperactivity and longer-lasting haloperidol-induced catalepsy but did not affect amphetamine sensitization and withdrawal and the reactivity to apomorphine and haloperidol in the prepulse inhibition (PPI) tests or to antidepressants in the haloperidol-induced catalepsy [96]. Consistently, Issy et al. [97] also found that injection of anti-EAG1 antibody into the dentate gyrus of hippocampus did not modify apomorphine-disruptive effects in the PPI response. However, it is noteworthy that the EAG1 antibody completely restored the startle amplitude decrease revealed after dentate gyrus surgery. More in-depth inspection on the EAG1-deficient mice using patch clamp and two-photon Ca^2+^ imaging by Mortensen et al. [20] demonstrated that EAG1 is enriched in the presynaptic terminals and is not involved in the somatic action potentials (APs) in the cerebellum. However, they found that the EAG1 channel regulates Ca^2+^ influx and neurotransmitter release during repetitive high-frequency activity. For example, the authors noticed that Ca^2+^ influx into axonal boutons was enhanced in mutant mice in response to the stimulation with three APs, but not to a single AP. This stimulation frequency-dependent Ca^2+^ release indicates that the EAG1 channel plays a fine-tuning role in synaptic transmission during high-frequency firing when other potassium channels suffer cumulative inactivation [20]. In addition, the presence of EAG1 channels in the nigrostriatal pathway in the rat brain indicates that EAG1 channels may be involved in Parkinson's disease characterized by partial or complete loss of dopaminergic neurons [98]. The observations by Ferreira et al. showed that both EAG1 channel and tyrosine hydroxylase immunoreactivity decreased in ipsilateral striatum in the neurotoxin 6-hydroxydopamine (6-OHDA) injected rats [98]. On the basis that the reactive oxygen species (ROS), such as the superoxide anion and H_2_O_2_, mediate the cytotoxicity of dopaminergic neurons by 6-OHDA [99] and EAG1 channels are greatly sensitive to H_2_O_2_ modification [31], it will be worth exploring the physiological roles of EAG1 channels in dopaminergic midbrain neurons as well as in Parkinson's disease.

## 4. EAG1 Channel Dysfunction in Genetic Neurological Disease

With the advance in the genome sequencing, the pathological functions of EAG1 channels in the nervous system are beginning to be revealed. A series of clinical observations have shown that gain-of-function of EAG1 gene mutations is strongly associated with two severe neurological and developmental disorders, Zimmermann-Laband syndrome (ZLS) [21, 22] and Temple-Baraitser syndrome (TBS) [23, 100]. The EAG1 mutations were also found in some undefined syndromes with intellectual disability and overlapping characters of ZLS and TBS [24, 50], which greatly increases the appreciation about the importance of physiological function of EAG1 channels. Here, we will review the recent literatures [21–24, 50, 100] and summarize the advances in the understanding of EAG1 channel mutations in the etiology of ZLS/TBS.

### 4.1. The Relationship between the Gain-of-Function of EAG1 Mutations and Phenotypes of ZLS/TBS

**Figure 2:**
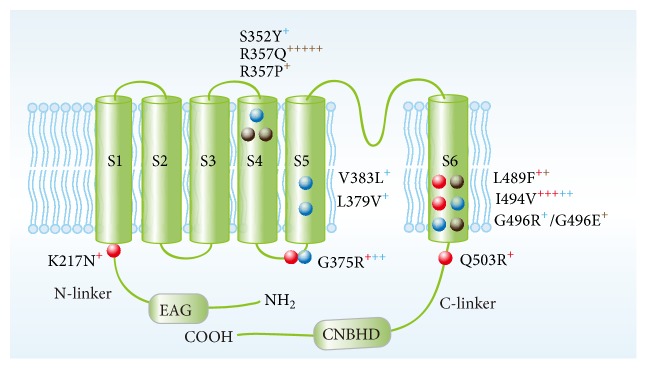
Schematic presentation of KCNH1 channel subunit (NM_172362.2) and the location of all identified mutations associated with TBS (red balls), ZLS (blue balls), and undefined neurodevelopmental disorders (brown balls). The amount of “+” indicates the number of patients with a certain type of mutations, and the color of “+” corresponds to patients with a certain type of disorders.

Although sharing some similar syndromes, TBS and ZLS patients show distinct features: the former is characterized by intellectual disability, epilepsy, and hypoplasia or aplasia of the nails of the thumb and great toes [101, 102], and the latter is characterized by gingival enlargement, intellectual disability, hypoplasia or absence of all nails and terminal phalanges, and hypertrichosis and associated with or without epilepsy [103–105], respectively. Using whole-exome sequencing (WES) technique, Simons et al. [23] and Kortüm et al. [21] almost simultaneously connected the genetic causes of ZLS and TBS to EAG1 channel gain-of-mutations. So far, a total of twenty-two cases with pathogenic EAG1 mutations have been reported in all six published papers [21–24, 50, 100]. Twelve different gain-of-function EAG1 mutations have been identified in twenty-two patients, in which five mutations are TBS-associated [23, 100], six mutations are ZLS-associated [21, 22], and four mutations are associated with atypical syndromes of ZLS/TBS as shown in Table 1 [24, 50]. However, no loss-of-function mutation was identified (Figure 2). Interestingly, three mutations including Ile494Val, Gly375Arg, and Leu489Phe were found to be associated with both ZLS/TBS and the patients with atypical syndromes [23, 50], suggesting a crossed genotype-phenotype correlation. Such a correlation is theoretically possible based on the fact that the specific clinical characteristics of ZLS and TBS have not been clearly classified yet, which will definitely make it difficult to diagnose the patients as TBS or ZLS. Generally, one can distinguish TBS from ZLS according to their characteristic phenotypes displaying the nail hypoplasia or aplasia only on the great toes and thumbs in TBS but all nails dysplasia in ZLS [101, 102, 106, 107]. But not all the patients always present these distinct phenotypes even when associated with the same EAG1 mutation such as Leu489Phe [23, 50]. In fact, some common clinical features including intellectual disability, epilepsy, deafness, and seizures can be noted among these neurodevelopmental disorders, showing the overlapping phenotypes [100].

Both Ile494Val and Gly375Arg mutations were detected in patients with ZLS and TBS [21–23, 100], providing the stronger evidence that these two syndromes belong to a phenotypic continuum. However, the careful clinical comparison has found that the patients carrying the same mutations still show various phenotypes. For instance, both the patient # 3 with ZLS (19 years old) in Kortüm et al.'s study [21] and that with TBS (9 months old) in Mégarbané et al.'s study [100] carried Gly375Arg mutation, but the seizures only occurred in patient # 3 with ZLS [21, 100]. On the other hand, even though we hypothesized that the EAG1 mutation is the major cause of ZLS/TBS, other factors could also play a tuning role in shaping the distinct phenotypes. It is worth mentioning that patient # 2 with ZLS from Kortüm et al. [21] carrying two mutations in* cis* displays nail aplasia of three toes which means the patient showed a more severe limb abnormality than other ZLS patients with only one site mutation [21]. It seems that a single point KCNH1 mutation is not enough to cause the distinguishable phenotypic clinical features for all patients. Hence, genetic mutation accumulation may contribute the serious phenotypic syndromes.

### 4.2. Is the Hotspot Mutation Indicated?

Identification of a hotspot mutation would simplify the clinical diagnosis for the prospective patients, offering a possibility of understanding the etiology of these neuronal disorders and even to find a potential treatment. However, more than half of the patients with the typical and atypical ZLS/TBS syndromes carry different mutations as shown in Table 1. Moreover, even the rest of the patients with the same genetic mutation clearly show different clinical phenotypes. Although Arg357 and Ile494 mutations have been identified in six and five different patients in ZLS and TBS, suggesting these two mutations maybe high frequently occurred and can be recognized as the hotspot mutations in ZLS and TBS, respectively, it still needs further clinical and genetic confirmation given a very small number of patients in the studies and the diverse phenotypes of patients carrying the same mutation [21, 23].

### 4.3. Functional Research of Gain-of-Mutation of EAG1 Channel and the Potential Correlation with Epilepsies

K^+^ channels are a major negative regulator of membrane excitability and impairment of K^+^ channel function by genetic mutations has been involved in multiple human epileptic [108, 109]. Although the causality between the cellular excitability and epilepsy is not demonstrated yet, the channel blockers of voltage-gated Na^+^ and Ca^2+^ channels, and channel activators of GABA receptors are therapeutically effective in a large amount of patients with epilepsy [22]. The epileptic syndrome, a very common character of ZLS/TBS patients, however, was found to be associated with the gain-of-function of EAG1 mutation. The electrophysiological function of the gain-of-function mutatuion of EAG1 channel has been tested in a heterologous expression system [21, 23]. The mutant channels displayed no apparent difference in the current amplitude compared to wild-type channels, but most of the mutants exhibited an accelerated activation and slower deactivation kinetics. The apparent threshold of voltage-dependent activation is also more negative than that of wild-type channels [21, 23]. The overall electrophysiological property changes indicate that the gain-of-function mutation would result in an enhanced K^+^ transporting function, which prefers to maintain a more negative resting membrane potential thereby limiting the opening of voltage-gated Na^+^ and Ca^2+^ channels, two major effectors during epilepsy [21]. This EAG1 mutation with gain-of-function compared to that in other K^+^ channels with loss-of-function indicates that the mechanisms mediating the epilepsy may be different from that in classical types of epilepsy [109], although some sodium channel blockers and GABA receptor agonists such as rufinamide, topiramate, and nitrazepam have been used to control seizures on the KCNH1 mutation-affected cases [22]. Interestingly, it has been noted that some gain-of-function mutations of K^+^ channels are also implicated in human epilepsies [110]. The following studies by Shruti et al. have indicated that the elevated firing activity in neocortical pyramidal neurons is a potential mechanism of epileptic patients associated with a gain-of-function mutation of the large conductance and Ca^2+^ activated K^+^ (BK) channel [111]. How the gain-of-function of EAG1 mutant channel leads to epilepsies remains elusive. Given both types of channels mainly expressed in presynaptic membrane of neurons [20, 111], the gain-of-function mutation of BK and EAG1 channels may share common mechanisms in epilepsy generation. Thus, in addition to the electrophysiological properties of the gain-of-function of EAG1 mutant channels on heterologous models, further investigating whether the mutant EAG1 channels change the overall electrical properties of the neurons in situ and in vivo models will be critical to elucidate the mechanisms of epilepsy associated with EAG1 channel mutation.

### 4.4. Potential Mechanisms of Gain-of-Function Mutation of EAG1 Channels

The specific mechanism by which these mutations change the gating behavior is not clear. The recent cryo-EM structural model of rEAG1 channel may provide some clues. The cryo-EM structure of rEAG1 channel has revealed that the S4 segment directly interacts with C-linker to induce a bent in S6 segment to close the channel under a hyperpolarized state and the bend will come back without the interaction with S4 to open the channel under a depolarized conformation [14]. Therefore, any changes on these helices would perturb their movements, destabilize the closed state, and affect the biophysical properties of EAG1 channels. To understand the potential mechanisms, the localization of all 12 mutations was indicated in Figure 2. which shows that some of the mutation sites are located in the S4, S6, or C-linker domains important for EAG1 channel activation. The mutation in these domains could perturb movement of these domains and the subsequent interaction, favoring the opening state. In addition, the cryo-EM structural model of rEAG1 has shown that S4-S5 linker can direct the cytoplasmic C-terminal end of S4 to move toward the C-linker by interacting with the C terminus of S6 under the channel open state. The spatial orientation and proper distance between the C terminus of S4 and the C-linker are necessary for their interaction when the voltage sensors are in the hyperpolarized state. Hence, Gly375Arg mutation is likely to obstruct the S4-S5 linker motion to the direction of C terminus of S4, which could influence the proper channel gating. Moreover, the S4-S5 linker can be an integrator of the N terminus of the PAS domain to modulate the activation kinetics of EAG1. Thus, any amino acid mutation in the linker could affect the movement of neighboring N terminus of PAS or the whole PAS domain thereby changing the activation kinetics of EAG1 channel. The mutation in the S4-S5 linker could also interrupt its interaction with the N terminus of PAS and may have an impact on the channel's activation kinetics, shifting of the gating equilibrium of EAG1 mutant channels to the open state. All these hypotheses will give insights into the molecular mechanism by which the mutations in these domains may have a gain-of-function. A recent report has shown that Arg327 mutation in EAG2 channel promotes the channel constitutively opening by reducing the interaction with other negatively charged residues [112]. The cryo-EM structure has suggested that a region formed by F261 and D264 from S2 and D299 from S3 can facilitate the traverse of arginine residues in S4 when the charge transfer center is moving outward with depolarization. Mutation Arg357, a residue located below the voltage sensors in S4, may impede the backward movement of voltage sensor from the activated state. The more detailed molecular mechanisms of the gating of the mutant EAG1 channels need further investigation.

## 5. Conclusion and Perspective

The fine-tuning effect of EAG1 on synaptic transmission in transgenic mice models may suggest that more in-depth investigation of differential phenotypes from the cellular to in vivo level is necessary. Indeed, besides the plasma membrane, EAG1 channel has also been reported to be present in the inner membrane of the nuclear, indicating the channel may play some unexpected roles in gene regulation. In addition, although multiple gain-of-function mutations of EAG1 channels have been implicated in the developmental neurological disorders including epilepsy, more mechanistic studies are badly needed to build a causality connection between the genetic changes of EAG1 and clinical phenotypes of neuronal disorders. All these efforts will greatly improve our understanding of physiological and pathological significance of EAG1 channels.

The gain-of-function mutation of EAG1 channels in ZLS/TBS and the aberrant overexpression of channels in cancer cells have strongly suggested that targeting EAG1 channels is ideal diagnostic and therapeutic strategies for both EAG1-associated neuronal and nonneuronal diseases. Unfortunately, no specific EAG1 channel blockers are available. The crystal structure of eag-CNBHD complex provides a promising approach for generating specific drugs targeting eag-CNBHD complex, the most distinct part of EAG1 channel compared to other Kv channels. However, pursuing for a specific EAG1 channel gating inhibitor by targeting the eag-CNBHD complex failed to find any high affinity leads [113]. The recent cryo-EM structural model of full-length EAG1 channel will facilitate the specific inhibitor discovery process. In addition, the research on endogenous regulations and the involved mechanisms will also be contributable. For instance, intracellular Ca^2+^/CaM and PIP_2/3_ have been shown strong inhibition on EAG1 channel gating. But the physiological significance caused by these gate inhibitors is largely unknown. The recent research has revealed that the dynamics of membrane phospholipids is regulated by membrane potential and intracellular local Ca^2+^ [112, 114], indicating that the EAG1 channels are located in a more complicated environment in which any changes in PIP_2/3_, Ca^2+^/CaM, or membrane potential may eventually change the EAG1 channel function. Understanding such a complicated regulation network will be helpful to further reveal the functions of EAG1 channels in both physiological and pathological conditions.

The findings that EAG1 channels are expressed in dopaminergic neurons strongly suggest that the channel may function in Parkinson's diseases and other types of neurodegenerative diseases in which the oxidative damage plays important roles. Moreover, the elucidation of mammalian EAG1 channel structure and the potential mechanisms for channel regulation by a variety of endogenous factors widely expressed in our body including nervous systems will definitely provide valuable information for EAG1 channel drug development.

## Figures and Tables

**Table 1:** Demographic, genetic, and clinical data in all reported subjects with KCNH1 mutations.

	Mutations	Reference	TBS	ZLS	Atypical
	615G>C Lys217Asn	Patient D Simons et al., 2015	+	−	−

	974C>A Ser325Tyr (Ser352Tyr) 1066G>C Val356Leu (Val383Leu)	Patient 2 Kortüm et al., 2015	−	+	−

	1070G>A Arg357Gln	Patient 1 Bramswig et al., 2015	−	−	+
		Patient 2 Bramswig et al., 2015	−	−	+
		Patient 3 Bramswig et al., 2015	−	−	+
		Patient 1 Fukai et al., 2016	−	−	+
		Patient 2 Fukai et al., 2016	−	−	+

	1070G>C Arg357Pro	Patient 3 Fukai et al., 2016	−	−	+

	1042G>A Gly348Arg (Gly375Arg)	Patient 3 Kortüm et al., 2015	−	+	−
		Patient Mégarbané et al., 2016	+	−	−
		Patient Mastrangelo et al., 2016	−	+	−

	1054C>G Leu352Val (Leu379Val)	Patient 5 Kortüm et al., 2015	−	+	−

	1465C>T Leu489Phe	Patient B Simons et al., 2015	+	−	−
		Patient 4 Bramswig et al., 2015	−	−	+

	1480A>G Ile494Val	Patient C Simons et al., 2015	+	−	−
		Patient E Simons et al., 2015	+	−	−
		Patient F Simons et al., 2015	+	−	−
		Patient 1 Kortüm et al., 2015	−	+	−
		Patient 6 Kortüm et al., 2015	−	+	−

	1405G>A Gly469Arg (Gly496Arg)	Patient 4 Kortüm et al., 2015	−	+	−

	1487G>A Gly496Glu	Patient 4 Fukai et al., 2016	−	−	+

	1508A>G Gln503Arg	Patient A Simons et al., 2015	+	−	−

Total	12/22	22	7	7	8

+: present; −: absent; TBS: Temple-Baraitser syndrome; ZLS: Zimmermann-Laband syndrome; atypical: undefined neurodevelopmental disorder. The mutations in brackets represent the translated mutations that are originally found in short KCNH1 isoform (NM_002238.3) and in long isoform (NM_172362.2) for the easier comparison.
